# Next-Generation Diamond Electrodes for Neurochemical Sensing: Challenges and Opportunities

**DOI:** 10.3390/mi12020128

**Published:** 2021-01-26

**Authors:** Erin K. Purcell, Michael F. Becker, Yue Guo, Seth A. Hara, Kip A. Ludwig, Collin J. McKinney, Elizabeth M. Monroe, Robert Rechenberg, Cory A. Rusinek, Akash Saxena, James R. Siegenthaler, Caryl E. Sortwell, Cort H. Thompson, James K. Trevathan, Suzanne Witt, Wen Li

**Affiliations:** 1 Department of Electrical and Computer Engineering, Michigan State University, East Lansing, MI 48824, USA; guoyue@msu.edu (Y.G.); saxenaak@msu.edu (A.S.); wenli@msu.edu (W.L.); 2 Department of Biomedical Engineering, Michigan State University, East Lansing, MI 48824, USA; thom1069@msu.edu; 3 Neuroscience Program, Michigan State University, East Lansing, MI 48824, USA; sortwell@msu.edu; 4 Institute for Quantitative Health Science and Engineering, Michigan State University, East Lansing, MI 48824, USA; 5 Fraunhofer USA Center Midwest, East Lansing, MI 48824, USA; mbecker@fraunhofer.org (M.F.B.); rrechenberg@fraunhofer.org (R.R.); jsiegenthaler@fraunhofer.org (J.R.S.); switt@fraunhofer.org (S.W.); 6 Division of Engineering, Mayo Clinic, Rochester, MN 55905, USA; Hara.Seth@mayo.edu; 7 Department of Biomedical Engineering, University of Wisconsin-Madison, Madison, WI 53706, USA; kip.ludwig@wisc.edu (K.A.L.); jtrevathan@wisc.edu (J.K.T.); 8 Department of Neurosurgery, University of Wisconsin-Madison, Madison, WI 53792, USA; 9 Department of Chemistry, Electronics Core Facility, University of North Carolina at Chapel Hill, Chapel Hill, NC 27514, USA; collin@unc.edu; 10 Department of Chemistry and Biochemistry, University of Nevada, Las Vegas, NV 89154, USA; rubioe4@unlv.nevada.edu (E.M.M.); cory.rusinek@unlv.edu (C.A.R.); 11 Department of Translational Neuroscience, College of Human Medicine, Michigan State University, Grand Rapids, MI 49503, USA; 12 Grainger Institute for Engineering, University of Wisconsin-Madison, Madison, WI 53706, USA

**Keywords:** diamond, neurotransmitter, FSCV, electrode, sensing

## Abstract

Carbon-based electrodes combined with fast-scan cyclic voltammetry (FSCV) enable neurochemical sensing with high spatiotemporal resolution and sensitivity. While their attractive electrochemical and conductive properties have established a long history of use in the detection of neurotransmitters both in vitro and in vivo, carbon fiber microelectrodes (CFMEs) also have limitations in their fabrication, flexibility, and chronic stability. Diamond is a form of carbon with a more rigid bonding structure (*sp*^3^-hybridized) which can become conductive when boron-doped. Boron-doped diamond (BDD) is characterized by an extremely wide potential window, low background current, and good biocompatibility. Additionally, methods for processing and patterning diamond allow for high-throughput batch fabrication and customization of electrode arrays with unique architectures. While tradeoffs in sensitivity can undermine the advantages of BDD as a neurochemical sensor, there are numerous untapped opportunities to further improve performance, including anodic pretreatment, or optimization of the FSCV waveform, instrumentation, *sp*^2^/*sp*^3^ character, doping, surface characteristics, and signal processing. Here, we review the state-of-the-art in diamond electrodes for neurochemical sensing and discuss potential opportunities for future advancements of the technology. We highlight our team’s progress with the development of an all-diamond fiber ultramicroelectrode as a novel approach to advance the performance and applications of diamond-based neurochemical sensors.

## 1. Introduction to Carbon-Based Sensors for Neurochemical Sensing

Disruption of chemical or electrical signaling in the brain underlies neurological disorders such as addiction [1,2,3], Alzheimer’s disease [4,5,6], amyotrophic lateral sclerosis [7,8,9,10,11], chronic pain [12,13,14,15], depression [16,17,18], Huntington’s disease [19,20,21], Parkinson’s disease [22,23,24], and schizophrenia [25,26,27]. Detection methods for sensing neurochemicals in vivo for the study of neurological disorders would ideally be simultaneously sensitive, minimally-invasive, chronically stable, and relatively inexpensive. In a recent review by S. Niyonambaza et al., techniques for neurotransmitter (NT) identification and quantification were discussed in depth [28], including positron emission tomography and single photon NT identification and measurement [29,30,31], single-photon emission computed tomography [32,33], surface-enhanced Raman spectroscopy [34,35], fast-scan cyclic voltammetry (FSCV) [36,37,38], amperometry [39,40], high performance liquid column chromatography (HPLC) [41,42,43], fluorescence [44,45], optical fiber sensing [46,47], and colorimetric measurements [48,49,50], as seen in Table 1. Longitudinal positron emission tomography (PET), while non-invasive, is not adequately sensitive to detect subtle changes in dopamine (DA) levels. While each available technique yields useful information, it is fast-scan cyclic voltammetry (FSCV) and HPLC coupled with microdialysis that allow for high temporal and spatial resolution for the detection of neurotransmitters (NTs) [51,52,53,54]. Of these two techniques, HPLC-coupled microdialysis can have a temporal resolution of up to 1 min by combining injection and analysis, separating and quantifying various NTs. Microdialysis is a powerful technique due to its sensitivity, selectivity, and number of simultaneous metabolites that can be separated and quantified [55]. Classically, however, microdialysis lacks spatiotemporal resolution because it has a relatively large probe diameter (~200 μm) and a typical sample collection time of every ~5–20 min [52,55,56]. Likewise, microdialysis probes are associated with pronounced glial encapsulation and disruption of blood vessels in comparison to small-diameter carbon fiber microelectrodes (CFMEs) traditionally used for FSCV [57]. CFMEs detected a ~90% decrease in DA concentration within the immediate vicinity of a microdialysis probe (~200 microns) in comparison to levels measured ~1 mm away following probe insertion [58]. The relatively large scale of the microdialysis probe may disrupt release and reuptake of the neurochemicals of interest. These results imply that the accuracy of NT detection may be influenced by the tissue damage caused by microdialysis probes, motivating the development of improved technology.

As an alternative, FSCV has been used to measure sub-second neurochemical signaling through electrochemical detection in situ [59]. FSCV has been widely used for real-time detection of NTs and other important bioanalytes, including oxygen (O_2_) and pH change. It provides improved spatiotemporal resolution compared to other short-time scale electroanalytical techniques such as chronoamperometry (CA). The technique uses an ultramicroelectrode with a small biological footprint (~7 μm in diameter) to produce a background-subtracted signal with high temporal resolution and nanomolar sensitivity [38,60,61,62,63,64,65,66]. FSCV involves two central steps: (1) adsorption of electroactive species of interest (e.g., neurochemicals) to the electrode surface is favored by the application of a small DC holding potential, and (2) a triangular voltage pulse is repeatedly swept across the interface to produce signature peaks in Faradaic current which result from oxidation/reduction of adsorbed neurochemicals. These peaks can be used to identify the specific neurochemical (based on the corresponding potentials) as well as its concentration (based on current amplitude). The typical voltage waveform that has been optimized to achieve selectivity, sensitivity, and stability for measuring DA with CFMEs is an applied potential swept from −0.4 to 1.3 to −0.4 V at 400 V/s, and reapplied at a frequency of 10 Hz [62,67,68]. Using this waveform and other developed waveforms, FSCV has been used to probe not only DA and serotonin (5-HT), but also other oxidizable neurochemicals, such as 3,4-dihydroxyphenylacetic acid (DOPAC), purines, ascorbic acid (AA), adenosine, norepinephrine (NEP), oxygen, pH changes, and hydrogen peroxide in vivo [38,60,61,62,63,64,65,66]. Improvements in selectivity and sensitivity have been achieved through further development of FSCV waveforms and application rate optimization. Development has also expanded NT measurement from phasic to tonic quantification and worked to increase technique safety and address biofouling effects on the chemical measurement [69,70,71,72,73,74].

The most commonly used materials for NT measurement with FSCV are carbon fibers due to their biocompatibility, electrochemical, and conductive properties [75,76,77,78]. CFMEs have been the cornerstone of in vivo FSCV and a bevy of data exist for the detection of electroactive NTs like DA, serotonin (5-HT), DOPAC, and others. Ralph Adam’s lab was the first to electrochemically measure DA in vivo using a carbon electrode consisting of graphite mixed with mineral oil [79]. Later, CFMEs were developed and used for dopaminergic and electrophysiological measurements in vivo, first published by Gonon [80], and then by Armstrong-James and Millar in 1979 [81]. Using the CFME, in 1981, Millar developed the technique of FSCV that was later popularized by Wightman [63,81,82,83]. A typical CFME consists of a carbon fiber that is aspirated into either a glass or silica capillary, or encased in some other insulating medium, such as parylene-C [74,84,85,86]. Electrodes also can be coated with polymers and other carbon-based materials to enhance the sensitivity and selectivity for various NTs [74,87,88,89]. Additionally, CFME surfaces can be functionalized with ease to tune the electrode to increase selectivity and decrease biofouling. Such coatings include: Poly(3,4-ethylenedioxythiophene) (PEDOT):Nafion [74], PEDOT:phosphorylcholrine [90], PEDOT: poly(ethyleneimine) (PEI) [88], CFME:gold nanoparticle [91], carbon nanospikes [89,92], Nafion carbon nanotubes [93,94], polycrystalline boron doped diamond [95,96], and carbon nanotube yarn [97,98]. Each coating has been tailored to not only increase sensitivity to NTs such as DA, but also to decrease the effects of biofouling and increase in vivo sensor lifetime. 

**Table 1:** Summary of neurotransmitter detection techniques.

Techniques	Advantages	Shortcomings	Reported LOD
PET	High spatial resolution	Complex manipulation Very high cost	Dopamine: 200 nM [99]
SPECT	High spatial resolution	Complex manipulation Very high cost	
SERS	Very high sensitivity and selectivity	Can be inapplicable in vivo depending on used material	Choline: 2 µM Acetylcholine: 4 µM Dopamine: 100 Nm Epinephrine: 100 µM
FSCV	High sensitivity	Low selectivity Electrode short lifetime	Dopamine: 50 nM
Amperometry	Low implementation cost	Low sensitivity and selectivity	Dopamine: 10 nM [100]
HPLC	High sensitivity and selectivity	High cost and complex manipulation	
Fluorescence	High sensitivity and selectivity	May not be usable in vivo	Dopamine: 10 pM
Chemilumin-escence	High sensitivity, and ease to couple with other methods	Indirect measurement through the loss of a signal due to a binding event	6 nM
Optical Fiber Sensing	High selectivity	Low sensitivity	Glutamate: 0.22 µM
Colorimetric	High sensitivity and selectivity, low cost	Not usable in vivo	Dopamine: 1.8 nM Noradrenaline: 20 µM Adrenaline: 2.5 µM

Table based on S. Niyonambaza et al. (reproduced from [28] under a Creative Commons Attribution 4.0 International License).

Despite their advantages, CFMEs have drawbacks which have motivated the search for alternative materials for in vivo FSCV. CFMEs are brittle and easily broken during insertion into the brain. Likewise, their long-term stability is compromised by dissolution of the carbon fiber electrode material that can result in significant degradation and loss of sensitivity over time. CFMEs are often fabricated through proprietary mechanisms, using low-throughput assembly methods, and are designed for industrial processes rather than electrochemical purposes [101]. Recently, boron doped diamond (BDD) deposition and growth processes were developed that enable the wafer patterning and growth of custom-deposited carbon electrodes [96,102,103,104,105]. Through these growth processes, BDD was grown on tungsten wires and carbon fiber surfaces. More recently, custom BDD microelectrodes (BDDMEs) encapsulated with polycrystalline diamond were developed [96,101,104,106,107]. BDD is an attractive material because it has a low background current, a wide potential window, and good biocompatibility [108,109,110]. Using BDD, Rusinek et al. showed that BDDMEs were suitable for neurochemical measurement using an all-diamond-electrode rather than the deposition of BDD onto another medium [103]. As carbon fibers are proprietarily fabricated, limiting the modification of the material for an optimized structure–function relationship, BDD electrodes are attractive due to the tunability of the carbon *sp*^2^ to *sp*^3^ ratio [111,112,113,114]. Increasing the *sp*^2^ character of the BDD increases the density of electronic states, and provides catalytic sites for redox reactions through adsorption sites. By using the BDD growth process, electrodes can be further tailored to enhance specific electrochemical properties. Such modifications include adjusting the structure–function relationship of the material to enhance conductivity, decrease capacitance, and increase chemical functionalization for selectivity and sensitivity. Additionally, recent advances in electrode array technologies for voltammetric measurements include multi-barrel glass capillary arrays [115,116], patterned arrays on silicon wafers [117,118], or parylene-C insulated multichannel carbon fiber electrode arrays [85,119]. While these arrays are powerful, most rely on hand fabrication processes under a microscope and are cumbersome, and slow. Open opportunities remain to improve the design and performance of carbon-based neurochemical sensors, including the development and optimization of diamond-based electrodes.

## 2. Motivation for Diamond Sensors for Neurochemical Detection

For FSCV, it is desirable for the electrode to display both high sensitivity (detecting minute concentrations of the neurochemical of interest) in addition to high selectivity (reliably differentiating the neurochemical of interest from interferents). Historically, the advantages of diamond electrodes have been counterbalanced by tradeoffs in sensitivity relative to CFMEs. While optimal translation of the positive in vitro performance characteristics of diamond electrodes to the chronic in vivo setting remains an unmet opportunity, there are advantageous characteristics of diamond which motivate further development.

### 2.1. Batch Fabrication and Customization

**Figure 1:**
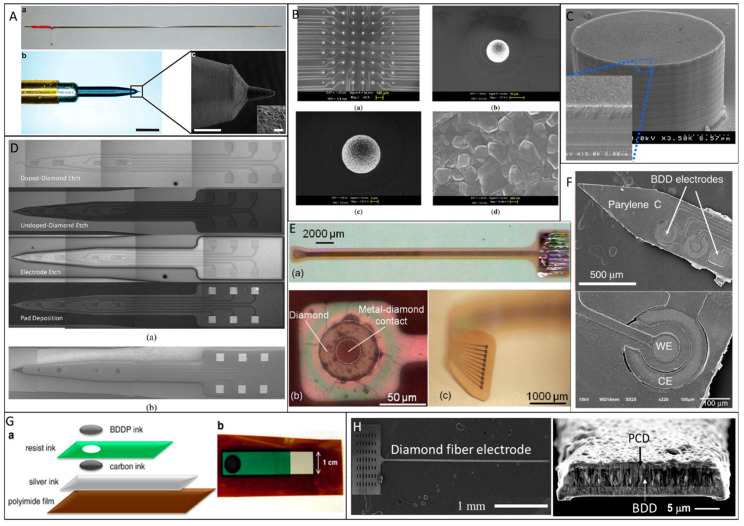
Examples of diamond-based sensors. (**A**) Polycrystalline boron-doped diamond (BDD) deposited on a conically-sharpened tungsten rods and insulated with parylene C (reproduced from [96] with permission *). (**B**) An 8 × 8 ultramicroelectrode array made of nanocrystalline BDD (reproduced from [126] with permission *). (**C**) Nanocrystalline BDD deposited on a pre-patterned high aspect ratio silicon pillar electrode (reproduced from [127] with permission). (**D**) All diamond microelectrode probe with polycrystalline BDD electrodes and traces insulated by undoped diamond thin films. (reproduced from [130] with permission *). (**E**) A flexible diamond-on-polymer electrode array fabricated by transferring pre-patterned polycrystalline BDD electrodes on metal-on-polynorbornene substrates (reproduced from [131] with permission). (**F**) Flexible diamond-on-parylene electrode probe in which the electrodes, traces, and contacts were all made of polycrystalline BDD (reproduced from [133] with permission *). (**G**) A screen printed diamond on polyimide electrode (reproduced from [136] with permission). (**H**) Our all diamond fiber electrode with a conducting BDD core encapsulated by a non-conducting PCD shell (reproduced from [103] with permission *). * Open access sources, reprinted under a Creative Commons Attribution 4.0 International License.

**Figure 2:**
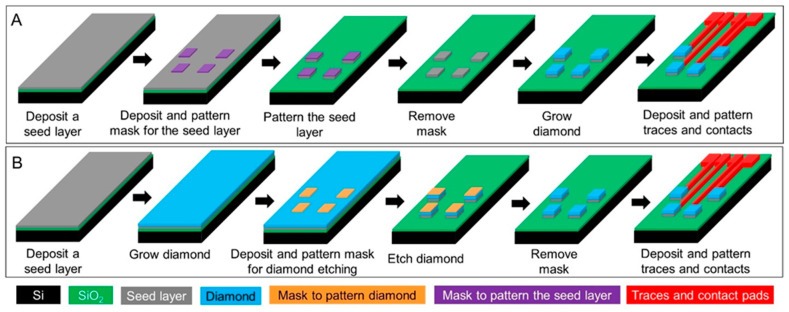
Schematic plots of the two typical microfabrication methods for making diamond microelectrode arrays. (**A**) The bottom-up approach where diamond electrodes are grown directly on a pre-patterned seed layer. (**B**) The top-down approach where a continuous diamond film is grown on the seed layer followed by diamond etching to form the electrode patterns.

While CFMEs often rely on manual assembly techniques that can be cumbersome and low-throughput, BDD electrodes can be patterned in a variety of architectures and batch-fabricated. To create BDD microelectrodes and multielectrode arrays (MEAs), several different approaches have been explored, which are typically divided into bottom-up approaches relying on chemical vapor deposition (CVD) growth of microstructures or top-down approaches by etching or machining BDD to produce microstructures [120]. A common approach to fabricate BDD microelectrodes in a probe format is the overgrowth of CVD BDD on the tip of sharpened and insulated metal (e.g., tungsten or platinum) microwires [96,121,122,123,124,125], followed by insulating the wire with pre-pulled glass capillaries [123,124], heated polypropylene pipette tips [125], or chemical vapor deposited parylene C (Figure 1A [96]). However, this approach has drawbacks. Exposing the same electrode geometry each time and up-scaling the device to high-density MEAs are the main challenges. To produce well-defined, high-density BDD MEAs, an alternative bottom-up approach has been demonstrated [126,127], wherein the diamond seed layer was pre-patterned prior to CVD growth, resulting in a patterned BDD film directly from the CVD process (Figure 1B [126] and Figure 1C [127]). In contrast, top-down methods grow a continuous BDD film on a seeded planar substrate (e.g., silicon or silicon dioxide wafers) followed by micromachining the BDD film to create microelectrode patterns via plasma dry etching [128,129] or laser ablation (Figure 1D [130]). Figure 2 shows schematically these two typical fabrication methods for making all diamond microelectrode probes and MEAs. To further improve the mechanical flexibility, pre-patterned BDD MEAs can be transferred from a solid substrate onto a soft polymer substrate, such as polynorbornene [131], polyimide [132] and parylene C [133,134] to form BDD-on-polymer MEAs with reduced mechanical rigidity (Figure 1E [131] and Figure 1F [133]). Recently, BDD paste electrodes suitable for electroanalytical applications have been reported by Kondo et al., offering a cheaper alternative to fabrication of flexible BDD MEAs [135,136] (Figure 1G [136]). These glucose sensing electrodes were constructed by screen printing of a glucose-oxidase-immobilized cobalt phthalocyanine/boron-doped diamond powders on a polyimide substrate. While these devices enable multi-channel sensing with improved spatial resolution, their large dimensions increase the risk of tissue damage and scar tissue formation. Our group has recently reported the fabrication of all-diamond, microelectrode fibers with a minimized footprint (Figure 1H [103]).

### 2.2. Wide Potential Window

**Figure 3:**
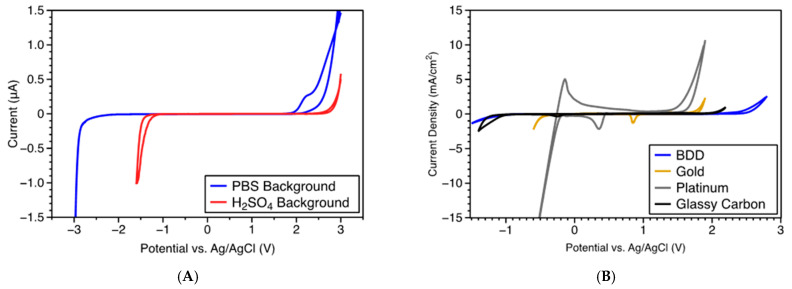
(**A**) Potential window of diamond fiber electrodes measured in pH 7.4 PBS buffer and 1.0 M H_2_SO_4_ (reproduced from [103] with permission as an open access source). (**B**) Comparison of the potential windows of a BDD macroelectrode and standard gold, platinum, and glassy carbon electrodes in 1.0 M H_2_SO_4_.

The electrochemical potential window is the potential range in which the electrode is stable without oxidation or reduction occurring in redox electrolytes. It is limited by the inherent electrochemistry properties of the electrode material and the redox behavior of the electrolyte [137]. When the electrolyte of interest is water, the electrochemical potential window is often known as the water potential window. BDD exhibits an extremely wide potential window in aqueous solution and quasi-reversible kinetics with low background current. Typical potential windows of 2.27–3.5 V have been reported for the BDD thin film electrodes [113,134,138,139]. As a demonstration of the wide potential window, we evaluated the electrochemical potential windows of BDD fiber electrodes in pH 7.4 phosphate buffered saline (PBS) and 1.0 M H_2_SO_4_ using cyclic voltammetry (CV), as illustrated in Figure 3. We used a three-electrode cell with the diamond fiber as a working electrode (WE), a platinum wire as a counter electrode (CE), and an Ag/AgCl electrode as a reference electrode (RE). The potential window was calculated from the cyclic voltammogram by subtracting the reduction potential (cathodic limit) from the oxidation potential (anodic limit). As shown in Figure 3A, the BDD fiber had a very wide potential window of ~5.0 V in pH 7.4 PBS and ~4.0 V in 1.0 M H_2_SO_4_ and low background currents over these potential ranges. The potential window of a ~3 mm diameter BDD macroelectrode is much wider than other common electrode materials, such as gold, platinum, and glassy carbon (Figure 3B). These properties are potentially desirable for applying BDD as an electrode material in electrochemical sensing. The wide potential window of BDD electrodes theoretically could enable the detection of a wide variety of chemicals with extended anodic potentials. Moreover, the very small background current of BDD electrodes can facilitate a low detection limit of analytes, provided that the decrease in noise enables an increase in signal-to-noise ratio (SNR).

### 2.3. Selectivity

Selectivity for FSCV measurement of neurotransmitters is achieved by assessing peaks in Faradaic current that result from electroactive species. However, the complex brain environment contains a variety of electroactive species that commonly produce overlapping Faradaic currents that complicate the discrimination of neurotransmitters of interest from interferent molecules. The relative contributions of different neurotransmitters to Faradaic currents recorded during FSCV depends not only on the reduction and oxidation potentials for each molecule, but also on the adsorption properties and reaction kinetics. Increased selectivity and specificity for specific neurotransmitters based on their adsorption and kinetic properties can be achieved by optimizing the waveform applied to an FSCV electrode. For FSCV at CFMEs, characterization of how changing waveform properties affects the representation of interferents has led to optimized waveforms for the measurement of specific neurotransmitters including DA [140], adenosine [141], and serotonin [37] (5-HT). For example, studies show that, by increasing the anodic potential limit of CFMEs from 1.0 to 1.4 V during a CV scan, increased sensitivity to DA was achieved [140]. The improvement in sensitivity is mainly attributed to an increase in the adsorption properties of the CFMEs. Unfortunately, etching occurs at potentials greater than 1.1 V for CFMEs, which can be advantageous for cleaning the electrodes to alleviate fouling [140,142], but also risks creating drift in the background. This overoxidation of CFMEs by scanning over an extended potential window could cause a continuous loss of electrode mass, possibly due to evolution of carbon dioxide [142,143]. In addition, the higher potential limit alters the electron transfer kinetics at the CFMEs, causing a decrease in temporal response and decreased selectivity for DA over interferents such as DOPAC, NE, and 4-hydroxy-3methoxyphenylethylamine (3-MT) [62,140]. With the extended anodic potential, the peak oxidation potential for DA became sharper and the sensitivity was enhanced by 9 ± 2 fold (in vivo) and 4.9 ± 2 fold (in vitro). The extremely wide potential window and stability of BDD electrodes may allow the anodic potential to be pushed to a higher limit to achieve better sensitivity of electroanalysis while maintaining selectivity. Additionally, stable scanning to extended anodic potentials enabled by the wide potential window may allow for measurement of additional molecules with oxidation potentials beyond traditional FSCV waveforms. Further, the extended scan range could potentially increase selectivity by enabling the measurement of additional oxidation peaks at extended potentials, such as the secondary oxidation peak for epinephrine that occurs at around 1.6 V due to the presence of a secondary amine that enables the differentiation of epinephrine and norepinephrine [38]. Thus far, the characterization of interferents at diamond electrodes has been very limited (Table 2), but will be an important factor in optimizing the selectivity of diamond electrodes. The tradeoffs with wide potential window, selectivity, stability, and temporal resolution described in this section should be carefully considered in the design and characterization of BDD electrodes.

### 2.4. Sensitivity

The use of electrochemical sensors in the complex brain environment requires high sensitivity and selectivity against other electroactive species that can interfere with the analyte of interest. For example, interferents, such as DOPAC, AA, and uric acid (UA), greatly hinder the electrochemical determination of DA in the brain, because these chemicals exhibit much higher concentrations than those of DA and oxidize at similar potentials to that of DA. Surface fouling due to accumulation of oxidized products on the electrode surface further limits electrode selectivity and sensitivity in the long-term. While challenges exist, the inherent robustness, electrochemical stability, wide potential window, and good electrocatalytic activity of highly boron-doped polycrystalline or nanocrystalline diamond electrodes make these materials suitable candidates to detect DA [96,105,133,144,145], epinephrine (EP) [146], and NEP [147,148]. These electroactive NTs belong to the family of catecholamines (e.g., DA), which are readily oxidized to an ortho-quinone that can be electrochemically detected using pristine BDD electrodes without any surface modification or functionalization. For example, Tyszczuk-Roko et al. tested BDD electrode for DA determination in biological samples and pharmaceutical preparation using CV and differential pulse voltammetry (DPV), and obtained a limit of detection (LOD) of ca. 187 nM with good linearity in a wide concentration range of 10 to 200 µM [144]. Most recently, our team pushed the DA LOD of untreated BDD fiber electrodes to ca. 30 nM by increasing the scan rate of FSCV to 900 V/s (see more details in the following section). Besides catecholamine NTs, 5-HT, an indolamine NT, contains the phenolic group, which undergoes an oxidation process leading to the formation of ketones. Electroanalytic sensing of 5-HT has also been carried out on unmodified BDD electrodes by several groups, with achievable LOD down to 10 nM [149,150,151]. A summary of the neurochemical sensing performance of the untreated BDD electrodes is reported in Table 2.

**Table 2:** Neurotransmitter sensing performance of untreated BDD.

Electrode	Linear Range/µM	LOD/µM	Sensitivity/µA µM^−1^	NTs/Interferents	Detection Method	Ref.
As deposited BDD	0.01–200	0.187	---	DA/Paracetamol	CV and DPV	[144]
As deposited BDD (fiber electrode)	0.05–10	0.03	---	DA	FSCV (in vitro) and Square wave voltammetry [SWV]	Our work
As deposited BDD	0.01–100	0.01	0.017	5-HT	CV	[149]
As deposited BDD	---	0.01	---	5-HT	FSCV (in vivo)	[152]
As deposited BDD	0.7–60	0.21	---	EP	SWV	[146]

Nonetheless, studies thus far suggest that untreated diamond has lower sensitivity to NTs of interest in comparison to CFMEs, perhaps in part due to lower adsorption. In some studies, demonstrations of the sensing performance of diamond-based electrodes are based on non-physiological, artificially high concentrations of DA. For example, in a comparison of CFMEs and ultrananocrystalline diamond microsensors (UNCD), a DA uptake inhibitor (nomifensine) was required to register a detectable DA signal on the UNCD electrodes following implantation into the rat striatum [153]. However, competitive detection limits to CFMEs were achieved through a combination of electrochemical pretreatment, UV exposure, and creating a similar form factor to the CFME. These modifications were suggested to reduce *sp*^2^ content, increase oxygen termination, and reduce the size of the electrode to be more comparable to CFMEs. While Kitazawa and colleagues observed reduced sensitivity of diamond electrodes in comparison to CFMEs, they noted that waveforms had not been optimized for diamond, and performance was comparable using constant amperometry [154]. Anecdotally, a recent observation from our team indicated that the CFME offered a ~14× increase in sensitivity in comparison to a BDD electrode, although it is notable that the BDD electrode was 27× smaller in surface area compared to the CFME and not quite as conductive (as indicated by an anodic shift of the DA oxidation peak). True 1:1 comparisons of the performance of CFMEs and diamond electrodes can be challenging, particularly considering differences in electrochemical surface area, as well as that waveforms and instrumentation typically used for diamond electrodes were originally tailored to CFMEs. Differences in the geometry of the electrodes can also play a role: electron transfer kinetics vary at the edges versus the tip of CFMEs. Likewise, assessments of background drift, biofouling, NT polymerization, and SNR should be included in comparisons of electrodes.

### 2.5. Biocompatibility

Material biocompatibility is an important consideration for implantable neural electrodes. Biofouling is a key determinant of sensitivity, and both protein and cellular adhesion have the potential to interrupt the performance of implanted sensors. Glial scarring and neuronal loss can contribute to noise and effectively isolate the sensor from target electrical and neurochemical signals [155,156]. The glial encapsulation can extend up to several hundred microns from the implant center [157], which is typically ~10–100× the electrode size. Glial encapsulation can present a diffusion barrier at the electrode interface, with the potential to significantly degrade the transformation of neurochemical signals to the electrode over time [155,158]. Diamond is a bioinert material with an established track record of success as a candidate implant material [159]. In addition to its attractive material properties (fracture toughness, corrosion resistance, etc.), diamond is recognized as non-immunogenic and non-thrombogenic [159], with demonstrated biocompatibility in both in vitro and in vivo settings [133,160,161]. For sensing applications, conductive, boron-doped diamond exhibits fibrotic encapsulation and inflammatory responses which are less than or equivalent to medical-grade silicone following implantation intramuscularly in guinea pigs [160]. Notably, there may be a relationship between the level of dopant and the degree of cellular adhesion, where increased cellular adhesion to more positive surface potentials resulting from increased dopant is a possible underlying mechanism [160]. In retinal implants, diamond is a promising candidate encapsulation material, lacking observable inflammation in tissue collected at the two week time point during a six month intraocular implantation period in rabbits [161]. Likewise, polycrystalline diamond is a biocompatible substrate for neuronal culture, illustrating similar neuritic arborization and outgrowth to control cell culture plastic [133].

### 2.6. Flexibility

While diamond has been recognized as a biocompatible material, its rigidity has the potential to exacerbate trauma to surrounding brain tissue, both during device insertion and through subsequent cyclical damage resulting from the natural pulsatile micromotion of the brain relative to a skull-fixed implant [157]. In the long term, the presence of stiff implants may contribute to inflammatory responses, resulting in neural degeneration and glial scar formation surrounding the implant. Fabrication of an electrode implant with small dimensions, which improves mechanical flexibility through reduced bending stiffness, is one solution to minimize the inflammatory response and prolong the functional lifetime of the device. In neural sensing applications, subcellular dimensions have been shown to facilitate a reduced tissue response to implanted electrode arrays [156]. Carbon-based electrodes with small feature sizes (7 micron diameter) have demonstrated minimal observable gliotic scarring and improved integration into surrounding brain tissue in comparison to traditional, silicon-based electrodes [162]. Diamond electrodes for neural sensing applications can be fabricated with similarly small device dimensions [103].

Bending stiffness, which is defined by both the device dimensions and the Young’s modulus of the material, has emerged as a critical determinant of biocompatibility [163]. By Euler’s formula, buckling force (*F_buckling_*) is defined as the force applied on a beam which causes the beam structure to fail, and determined by the beam’s width, height, length, and Young’s modulus of the beam’s material as indicated in Equation (1):

(1) $$F_{buckling}=\frac{\pi^{2}E\frac{wh^{3}}{12}}{{\left(Kl\right)}^{2}},$$

where *E* is the Young’s modulus of diamond (10^5^ GPa). Although diamond is stiffer than most materials, a diamond fiber can have a bending stiffness as low as some polymer [164] and carbon fiber [85] probes when the cross-sectional dimensions of the fiber are small relative to the fiber length.

According to the theoretical modeling and finite-element analysis conducted in [157], the substrate material should be as soft as possible to effectively integrate with brain tissue. However, many “soft” materials, such as polydimethylsiloxane (PDMS), parylene-C, and other materials with Young’s modulus smaller than 10 GPa [165] have limitations, or in practice, “impossibilities” for applications in neural implants. For example, the fabrication of such soft probes usually requires unconventional and tedious processes, and the surgical insertion of the device to the target tissue requires temporary or permanent sheaths to reinforce the mechanical rigidity and avoid buckling. Additionally, when the probe shank is too soft as discussed, micromotions of the brain can cause displacement on electrodes [157], resulting in inaccurate measurements from the same neurons. As such, an ideal implantable electrode probe should be flexible and small to alleviate inflammation response and tissue damage but mechanically stiff enough to facilitate device implantation to the tissue of interest while preserving the implant from being broken in the brain environment [156,166]. Because diamond can be fabricated in subcellular dimensions, the device can retain competitive bending stiffness while retaining adequate rigidity for insertion.

## 3. Opportunities for Optimization

The performance of diamond electrodes remains a challenge in the in vivo setting, where interferents and biofouling can undermine the advantages of the wide potential window to selective detection of neurochemicals of interest. Much fundamental work remains to be completed in order to properly characterize and optimize neurochemical detection with BDD electrodes, where surface modifications, waveform optimization, and instrumentation tailored specifically to BDD electrodes are key opportunities to meet remaining challenges.

### 3.1. Instrumentation

When comparing the performance of the CFME and diamond electrodes, it should also be considered that the instrumentation (e.g., universal electrochemistry instrument (UEI) built by the University of North Carolina Electronics Design Core Laboratory), software (e.g., high definition cyclic voltammetry software (HDCV), University of North Carolina, Chapel Hill, NC, USA), and flow cells used, are typically specifically engineered for the CFME [167]. All of these components have been designed considering the CFME electrode surface area/geometry, magnitude of the capacitive background current, and overall electrode conductivity. Future iterations of BDD electrodes will also include changes to the instrument hardware and software to better accommodate their specific properties. Specifically, attributes such as headstage gain and capacitance compensation, either by electronics or analog background subtraction, will be optimized for various BDD electrode active areas. To accommodate BDD arrays, the hardware will be expanded to include large channel capabilities, such as 16, 32, and 64 channels, requiring a shift in paradigm from using a PC (personal computer) to manage data collection to using an FPGA (field programmable gate array) to perform data collection at high speeds along with SOC (system on chip) integrated circuitry. In addition, since BDD active areas can be precisely controlled, electrodes with small areas can be used for probing transient electrochemical events [168]. This requires reductions in stray capacitances involving the method of connection with the electrode as well as modifications to the front-end electronics for improvement in bandwidth. Increases in instrumentation bandwidth can also facilitate the use of electrochemical impedance spectroscopy (EIS) for monitoring electrode health for extended operating times and the optimization of fast arbitrary waveforms for convolution-based background subtraction [169]. Presently, the UEI instrumentation is capable of performing simultaneous FSCV and electrophysiological measurements on four CFM electrodes. The circuitry to accomplish this with BDD arrays, which are also capable of single unit recordings, will need to be expanded.

### 3.2. Waveform

As noted previously, pure BDD has been shown to have an expanded water window compared to platinum or carbon fiber, demonstrating that specific reactions such as water electrolysis occur at different electrode polarization voltages. Similarly, the oxidation peak for DA has been shown to shift, depending on the study, from the typical 0.6 V for carbon fiber to ~1.0 V for more pure diamond. The potential limits and scan rates of the waveform used can influence the surface concentration of a NT, as well as the sensitivity and selectivity of the measurement [63]. For example, a negative potential applied between scans is used to concentrate catecholamines to the electrode surface. Several strategies have been employed to tailor waveforms to optimize the detection of specific analytes of interest, including deviations from the standard triangular shape (e.g., ‘sawhorse’), as well as within-scan changes in scan rate and amplitude of the sweep sections composing the overall waveform. The waveform also can be designed to promote release of adsorbed products on the surface of the electrode, alleviating the effects of biofouling and polymerized neurochemicals. In essence, the change in material requires, at a minimum, a re-optimization of the FSCV waveform to enhance sensitivity to a specific neurochemical of interest, as well as understand selectivity versus potential interferents.

### 3.3. Sp^2^/Sp^3^ Content

Through the fabrication process for BDD, both *sp*^2^-hybridized and *sp*^3^-hybridized carbon may be formed. *Sp*^2^-hybridized carbon is non-diamond carbon, such as graphite, whereas a diamond crystal lattice is formed from *sp*^3^-hybridized carbon. Opportunities to refine the sensitivity and selectivity of diamond electrodes include tuning *sp*^2^ carbon/impurity contents, surface morphology, and surface chemistry of BDD. Increased *sp*^2^ character decreases the stability of the material but also provides sites for neurotransmitter adsorption. Long-term benchtop studies of BDD performance over time need to be conducted to understand the stability of the capacitive and redox currents; both the slow loss of *sp*^2^ carbon and the inability to ‘refresh’ the surface of pure diamond electrodes in the presence of biofouling may dramatically impact sensitivity and/or selectivity over time. Generally, *sp*^2^-bonded carbon promotes outer-shell or inner-sphere electron-transfer mechanisms, reduces the potential window, increases background current, and increases undesired adsorption of chemical species [170,171,172]. On the other hand, as-grown BDD surfaces are usually hydrogen-terminated and display relatively fast electron-transfer kinetics and are electrically conducting via an unusual surface transfer doping mechanism [173,174,175]. These surfaces can be further refined using post-synthesis treatments such as alumina polishing, oxygen plasma, electrochemical oxidation, and chemical functionalization to fit end-user needs [176,177,178,179,180]. BDD surfaces terminated with oxygen functional groups (C=O, C–OH, COOH) exhibit low surface conductivity and sluggish kinetics, but can limit the impact of anionic interferents due to a lack of adsorption [173,174,179].

### 3.4. Surface Characteristics

Improving sensitivity of BDD electrodes also may be possible by increasing the electrochemically active surface area for a given lateral dimension by increasing the fractal dimension of the diamond electrode surface. This would provide more surface area for adsorption and mass transport of NTs. However, increased surface area increases the non-Faradaic background current. In some cases, the increased background currents can be large enough to saturate the input amplifier, in which case analog background subtraction can be applied using a UEI instrument with HDCV software and subtracting headstage [181]. Besides electrochemically treated BDD, porous BDD offers unique advantages including the large active area, strong surface adsorption, and high electron transfer properties [182], making it a good electrode material for highly sensitive and selective NT detection. Porous BDD can be created using plasma consisting of equal portions of H_2_ and Ar [183], or nanodiamond seeded SiO_2_ nanofiber templates [182], or vertically aligned carbon nanotubes scaffold [184,185]. Similarly, May et al. fabricated high-surface-area BDD electrodes using “black silicon”, a synthetic nanostructured material that contains high-aspect-ratio nano-spikes or needles, as a template during BDD synthesis [186]. It is yet to be determined whether or not these approaches will lead to long term improvements in the presence of biofouling and the reactive tissue response to the implant.

### 3.5. Methods for Improved Selectivity

**Figure 4:**
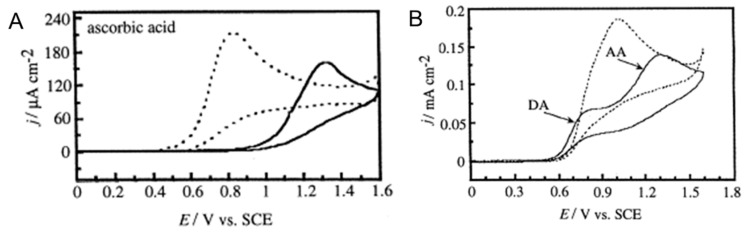
(**A**) Cyclic voltammograms for 1 mM AA at untreated (dashed lines) and anodically treated (solid lines) diamond electrodes in 0.1 M HClO_4_ solution. Sweep rate, 100 mV s^−1^. (**B**) Cyclic voltammograms for a mixture of 0.1 mM DA and 1 mM AA at untreated (dashed line) and anodically treated (solid line) diamond electrodes in 0.1 M HClO_4_ solution. Sweep rate, 100 mV s^−1^. (Reproduced from [187] with permission).

While interferents pose a challenge to selective detection of a NT of interest, separation of signature redox peaks can be enhanced through anodic pretreatment. As a proof-of-principle, it has been found that extensive anodic polarization in alkaline solution improved the selectivity of BDD electrodes towards DA in the presence of large excesses of AA [186,187], possibly due to the formation of oxygen-containing functional groups. The anodic treatment was carried out by holding the electrode potential at +2.4 V (vs Ag/AgCl) for 60 mins or at +2.6 V (vs SCE) for 75 min in 0.1 M KOH. The anodically treated diamond electrode effectively shifted the oxidation peak potential of AA to higher potentials while having minimal impact on the DA oxidation peak, resulting in large peak separation in CV scans (Figure 4). Recently, selective determination of NEP in the presence of lidocaine (LID) was demonstrated by Pınar et al. [188] using the BDD electrodes that were anodically pretreated in 0.5 M H_2_SO_4_ at +1.9 V (vs Ag/AgCl) for 180 s. High selectivity for DA in the presence of AA also can be achieved by surface hydrogenation of BDD electrodes via electrochemical cathodic treatment or plasma hydrogenation [189]. Hydrogen-termination increases AA oxidation kinetics and surface absorption, resulting in the downshift of the AA oxidation peak current to low potential. Surface modifications can cause surface morphology change of the BDD electrode, and the anodic treatment may also have a deactivating effect.

Surface functionalization using nanocomposites and polymers is an alternative strategy to improve the selectivity and sensitivity of BDD electrodes. Among different nanomaterials, nanoparticles (NPs) like carbon black (CB) and gold NPs exhibit excellent conductivity for fast electron transfer and a large number of defect sites, providing enough electrochemically active surface area. Moreover, negatively charged nanomaterials, such as AuNPs/polyelectrolyte-coated polystyrene (Au/PE/PS) colloids, are found to have high electrocatalytic activity that promotes the oxidation of DA while inhibiting the electrochemical reaction of AA, thus enabling selective determination of DA in the presence of AA with good sensitivity [190]. Such NP-based materials can be applied on the BDD surface in the forms of self-assembled monolayers (SAM) [191], self-assembled multilayer stacks [190], electropolymerized nanocomposite [192], or coating of NP-polymer suspension [189]. On the other hand, attempts also have been made to functionalize the BDD surface with polymers for selective detection of DA. For example, poly(N,Ndimethylaniline) (PDMA) coated BDD electrodes show a large negative shift (ca. 0.25 V) of the AA oxidation potential and an increase in peak current resulted from the favorable electrostatic attraction between the cationic PDMS film and anionic AA [193]. Likewise, Shang et al. modified the BDD electrode with an electropolymerized PTy/PPA film that has inherently high permselectivity of DA against 3,4-dihydroxyphenylalanine (L-DOPA), AA, uric acid, and other DA metabolites [194]. Table 3 summarizes the neurochemical sensing performance of BDD electrodes modified using different strategies.

A contaminating signal due to changes in pH is an important consideration for the use of BDD electrodes to detect NTs in the intact brain. It has been previously reported by Takmakov and others that CFMEs are sensitive to pH change through surface electrochemistry involving a hydroquinone moiety [66]. They observed multiple peaks in their FSCV response as pH was changed, primarily stemming from hydroquinone to quinone (and back) transitions. In an assessment of these impacts on BDD electrode performance, a recent observation from our group showed a normalized sensitivity that was 3–7× lower for diamond than that obtained with a CFME previously. Thus, there may also be hydroquinone moieties on the BDD surface which are responding to the pH change, but perhaps at a significantly less amount than that of a CFME. This result bodes well for the BDD electrodes as local pH changes in an in vivo measurement would seemingly not affect the capacitive background current as much as a CFME. Nonetheless, pH changes remain a significant challenge in the in vivo environment (more so than other interferents such as AA, which change on a slower time course), and potential optimization strategies to improve selectivity (Table 3) also should seek to mitigate these effects.

### 3.6. Stability

For carbon-based sensors, thermal stability decreases, the molecular adsorption (fouling) increases, and the mechanical hardness decreases with increasing non-diamond *sp*^2^ content [96,196]. Kalish et al. found that a diamond film with over 80% *sp*^3^ content is stable under a wide temperature range of 300–1270 K while a 40% *sp*^3^ film starts graphitizing at 700 K [197]. The surface termination is an additional factor that affects the electrochemical stability of a BDD electrode. As-deposited BDD usually has a non-polar, hydrogen-terminated surface due to film growth in a hydrogen-rich environment, thus reducing the adsorption of polar molecules [198]. However, this is controversial since the hydrogen-termination is very unstable in air. Nevertheless, high-quality BDD with hydrogen-termination and *sp*^3^-bonded carbon holds promise for suppressing the fouling effect and promoting the long-term stability of electrochemical analysis. Several studies show that the BDD electrodes exhibited excellent chemical stability even after months of exposure to biological and chemical environments [96,133,199,200,201,202]. For example, Rao et al. studied the stability of the BDD film electrode for nicotinamide adenine dinucleotide (NADH) in aqueous solution. The electrode response was reproducible for up to three months without any specific pretreatment [200]. Park et al. also found that the unmodified BDD electrode provided a highly stable response for exogenous NE in tissue for up to 7 h with response attenuation of <8%, much better than that of a bare CF electrode (>30%) [201]. While these studies are encouraging, further work is needed to explore translation of these benefits from the benchtop to the in vivo environment; it is important to verify that improved sensitivity does not come at the expense of altered selectivity.

### 3.7. Diamond Processing and Boron Doping

The performance of diamond electrodes can be further fine-tuned through the optimization of processing parameters. Diamond films may be grown using microwave plasma enhanced or hot-filament chemical vapor deposition (CVD), where gas phase carbon is deposited onto a substrate under certain pressures and temperatures. Boron doping of diamond materials may be accomplished by the introduction of diborane (B_2_H_6_), trimethylborane (B(CH_3_)_3_), or organic borates in the gas phase during the growth process, which alters the resulting film morphology, structure, and electrical properties [203]. Increasing the ratio of boron to carbon in the feedgas results in higher boron doping concentrations, and subsequently higher electrical conductivity of the film [172,204,205]. The transition of BDD from semiconducting to metal-like behavior occurs at a known boron concentration of 4–5 × 10^20^ atoms B cm^−3^ [206,207]. For electroanalytical sensing applications, higher boron doping concentrations typically lead to higher sensitivity and lower limits of detection [112,172]. This is due to increased charge carrier availability and faster charge transfer kinetics at the electrode surface for the oxidation of organic species compared to lowly doped BDD [112].

However, increasing the boron doping concentration can also decrease the potential window of BDD electrodes [172], which limits the applicability of highly doped BDD as an electrochemical sensor for certain analytes [112]. Furthermore, heavily doped BDD (>10^21^ B atoms cm^−3^) can contain added *sp*^2^-bonded carbon impurities that decrease the stability of the material [205,206,208]. These *sp*^2^-bonded carbon sites can also act as an adsorption site for reactants, which can catalyze redox reactions for species such as DA [112,172,206]. This may explain why smaller grain sizes typically result in higher electrochemical activity due to the prevalence of *sp*^2^-bonded carbon at the grain boundaries, as noted above [203]. With this in mind, an optimum boron doping concentration and *sp*^3^*/sp*^2^ ratio can be selected for any particular electroanalytical sensing application, taking into consideration the specific sensitivity, potential window, and material stability requirements. For example, in small molecule sensing where high sensitivities are required, a boron concentration of ~8000 ppm is commonly used [209,210,211,212,213,214,215,216,217,218,219,220,221,222,223,224]. This mid- to high-doping concentration yields a material with high conductivity and moderate *sp*^2^ impurities compared to heavily doped BDD [112]. Likewise, for neurochemical analysis, it is the increased sensitivity and fast electron transfer kinetics of highly doped BDD that makes it suitable for detection of DA and other NTs [105,145,187,225]. When a wider potential window is required, the usage of BDD electrodes with lower boron concentrations may be used, albeit at the cost of decreased sensitivity [112]. Based on the specific considerations for each application, the appropriate electrode properties may be determined, and BDD films with these optimal characteristics may be grown by adjusting the CVD conditions described above. The flexibility in the BDD growth parameters allows for the synthesis of films with a wide range of electrical and physical properties, and gives rise to the multitude of applications for BDD electrodes. For instance, while increasing boron doping concentrations also decrease the film grain size [172], larger grain sizes at higher boron doping levels may be achieved by increasing the reaction pressure [204]. By adjusting film growth parameters, such as pressure, power, temperature, feedgas composition, and growth time, the properties of the BDD film may be tuned to fit the end use case.

### 3.8. Reference Electrodes

In vivo FSCV in animal models is normally performed as a two-electrode electrochemical measurement using a small CFME and a larger Ag/AgCl reference electrode. Maintaining accurate applied voltages at the working electrode requires that the reference electrode is not polarized, or pulled away from its open circuit potential, over the course of a scan. This is achieved, in practice, by using a reference electrode with a much larger surface area, compared to the working electrode, as well as by using Ag/AgCl, which has low polarizability due to reversible reactions with chloride in solution. Although, Ag/AgCl reference electrodes have become the de facto standard, they pose a problem for long-term FSCV recordings due to degradation of the AgCl coating as well as adverse tissue reactions, as shown in chronic in vivo applications. These tissue reactions are characterized by extensive microglia encapsulation when the electrode is implanted within the CNS and fibrous tissue encapsulation in the periphery. These problems have been well known for decades. However, few alternative referencing strategies for FSCV have been explored. While avoiding the potential toxicity of silver in vivo, stainless steel is problematic due to ready polarization. While BDD electrodes have been proposed for long-term FSCV applications, appropriate reference strategies are an often overlooked aspect of a complete recording system. For long-term neurochemical recordings with diamond electrodes, it will be critical to ensure biocompatibility of the implanted reference electrode and the use of a reference electrode that has a stable potential over the course of a long-term implant.

### 3.9. Signal Processing

There are several challenges related to in vivo signal processing which have been established for carbon-based electrodes, and these challenges likewise will need to be considered for FSCV using diamond electrodes. The first step of FSCV signal processing typically involves the subtraction of the background charging current, which has a much larger amplitude than the Faradaic currents of interest. The background charging current is affected by the scan rate as well as the electrode characteristics [63,68,226]. Changes in the environment, temperature fluctuations, or changes to the recording surface itself can influence this reference measurement. In an in vivo setting, there are added factors that make the background subtraction even more unpredictable, disallowing a constant reference for subtraction from the voltammogram. A convolution-based method has been proposed as a potential solution for removing the background charging current [227]. In this method, the applied waveform and carbon fiber type are first optimized to simplify the background currents to components that can be predicted by convolution. A small voltage pulse applied immediately before each FSCV sweep is used to probe the electrode impedance and estimate its impulse response function through discrete differentiation. Convolution with the FSCV waveform allows for prediction and digital subtraction of the non-Faradaic background signal. On the other hand, it may be possible to extract useful information from the background current; information contained in the background charging current has been used to predict electrode sensitivity to sources of interference or the analyte of interest [63]. Another approach uses the oxidation potential of quinone-like moieties in the background signal as a predictor for the oxidation potential of DA [228]. Diamond is a material which generally exhibits lower capacitance and background currents than CFMEs, and further work is needed to test and optimize approaches to extract and assess the background signal with diamond electrodes.

Discrimination of the target NT of interest from interferents is an added challenge in the in vivo setting. Principal component regression is a technique which has been used to improve analysis of FSCV data collected. It performs the task of identifying the redox peaks of the NT from all the voltammograms (~1000 s) collected using a combination of principal component analysis (PCA) and multivariate regression. PCA applied to data collected across the voltammograms is used for training a machine learning algorithm. By comparing the principal components of the training set and the new data, the peaks of the analyte of interest are identified. However, this method fails when discriminating signals of similar variance. Another method known as the projection of latent structure (PLS), or partial least square regression (PLSR), performs a similar task to PCR. Here, PCA is performed twice: once for the predictor variables (voltammograms) and once for the response variable (corresponding concentration values). The subsequent analysis maximizes the covariance between the predictor and response variables. PLS is also more robust towards multicollinearity which happens when the predictor variables are highly correlated (i.e., individual current measurements in the voltammograms). In a comparison of the methods of processing, PLSR yielded higher accuracy and improved selectivity in the estimation of neurochemicals in both in vitro and in vivo data analysis [228]. Another drawback of these approaches is the need to obtain a separate training set for model generation, which can result in prediction errors if the training set is not carefully constructed from consistent data measured within the same animal with the same electrode. Multivariate curve resolution-alternating least squares (MCR-ALS) has been discussed [229] as an alternate approach to deal with this issue, which uses raw data itself to define component spectral and concentration profiles needing only the definition of number of components. Other considerations are the processing time for data analysis and errors due to bias. More recently, a novel image analysis technique was applied to color plots to successfully discriminate simultaneous DA and adenosine signals [230]. There are open opportunities to apply newer signal processing techniques to optimize NT detection with diamond electrodes, particularly in the in vivo setting.

Notably, it has previously been established using CFMEs that motion, RF noise, and electrical stimulation can generate an artificial signal in-vivo during FSCV that can closely resemble a phasic change in DA or adenosine [231]. The extent to which these artifacts resemble neurochemical changes may functionally change depending on the electrode material selected. Similarly, it is unclear for BDD electrodes if loss of *sp*^2^, DA polymerization to the surface, and/or biofouling will impact selectivity (the ability to distinguish the signal of interest from interfering signal such as pH change or oxygen change) in addition to sensitivity. These reasons underscore the need to optimize signal processing algorithms to establish selectivity in chronic use situations for new BDD FSCV materials.

## 4. Boron-Doped Diamond Microelectrodes (BDDMEs): A New Approach

Building on the state-of-the-art, our team is currently developing an all-diamond, BDD fiber electrode for neurochemical sensing (Figure 1H [103]). The devices can be batch-fabricated in customizable dimensions and geometries, as previously described [103]. We recently further simplified the fabrication process of the BDD fiber, taking advantage of the conformal nature of the CVD method. In this approach, a continuous BDD film is chemical vapor deposited on a seeded silicon wafer and patterned with a Ti/Cu mask to form the BDD fiber and contact. After dicing the wafer into small dies of devices, the silicon substrate under the fiber shank is chemically etched to form a suspended structure while the silicon under the contact region remained intact. In this case, silicon etching was conducted by dipping the tip region in an HNA solution (acetic acid:nitric acid:hydrofluoric acid = 55:35:20). Then microwave plasma assisted chemical vapor deposition (MPACVD) was performed to grow microcrystalline PCD conformally over the BDD structure, followed by laser cutting or mechanical cleavage to expose the recording site at the fiber tip. Finally, the silicon substrate under the contact region is completely removed to release the fiber and expose the contact pad. This simplified method requires only one lithographic mask, and therefore, improves the fabrication precision by eliminating the misalignment between masks. The pre-patterned Ti and Cu mask could be also utilized as a shadow mask to create BDD fiber patterns directly from CVD growth, since diamond growth on Cu is much slower than that on the seeded substrate.

**Figure 5:**
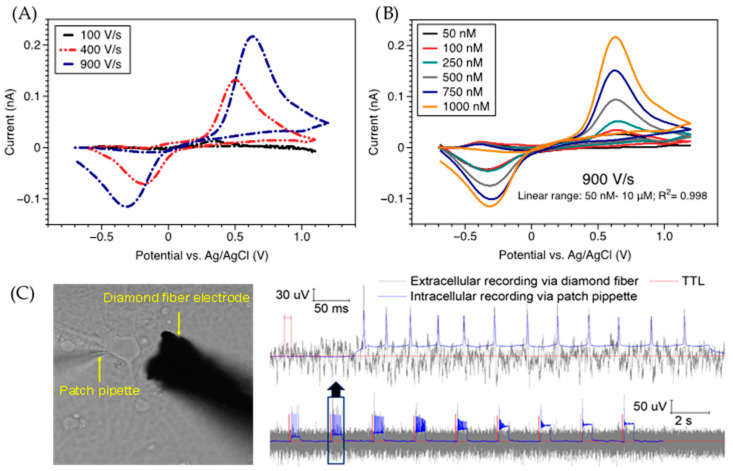
Data illustrating proof-of-concept of boron-doped diamond microelectrode (BDDME) performance as a sensor for neurochemical and electrical signals. Fast-scan cyclic voltammetry (FSCV) measurements validate the ability of diamond electrodes for rapid dopamine (DA) detection with good sensitivity and selectivity: (**A**) Scan rate study of 1.0 µM DA using FSCV, color plot inlay for 900 V/s (100–1000 V/s investigated). (**B**) DA FSCV calibration at 900 V/s and 10 Hz. Linear range: 50 nM–10 µM, R2: 0.998 (exemplary data shown). All experiments were completed in a stagnant cell containing pH 7.4 PBS. (**C**) Combined intra/extracellular recordings validate detectable spiking activity using the BDDME during stimulation through a patch pipette electrode.

Figure 5 shows data from our group which displays the chemical and electrical sensing capabilities of BDD microelectrodes (BDDMEs). Recently, we also successfully demonstrated the ability to use unmodified BDD fiber electrodes for ex vivo DA detection at a fast speed (900 V/s) and with high sensitivity (30 nM), linearity, and specificity. Using a three-electrode headstage, we performed FSCV to measure the sensitivity of DA using a diamond fiber WE, platinum CE, and a Ag/AgCl RE. We found that an increase in scan rate effectively improved the sensitivity towards DA, resulting in a 2× increase in oxidation peak current at 900 V/s (Figure 5A). Using 900 V/s, 10 Hz, and a slightly modified potential waveform (−0.7 to +1.2 V vs. Ag/AgCl), we calibrated the sensing performance of the BDD fiber electrode for low DA concentrations (Figure 5B), and observed good linearity from 50 nM–10 µM (R^2^: 0.998). We detected a clear oxidation peak of DA at 50 nM and obtained a limit of detection (LOD) of ca. 30 nM that is comparable to those of existing DA sensing electrodes made of CF or glassy carbon [74,232]. In addition to sensing neurochemical signals, BDDMEs are capable of detecting electrical signals generated by neurons (Figure 5C). To establish proof-of-principle, a patch clamp electrophysiology rig was used to both stimulate and record trains of action potentials from cultured rat cortical neurons using methods previously described [133]. Simultaneously, a BDDME placed in close proximity to the neuron was used to record the extracellular potential using a separate board (Intan), where signals were timelocked via a 5 V TTL pulse from the patch digitizer. Spikes in the extracellular potential were detected coincidently to intracellularly-recorded action potentials (“ground-truth” data). While subject to further optimization of the signal-to-noise ratio, these data demonstrate the ability of the BDDME to detect neuronal spikes in the extracellular potential.

The high-throughput, customizable fabrication scheme, bending flexibility, small footprint, and multi-modal sensing capabilities are key advantages of BDDMEs as a novel approach to diamond-based neurochemical sensors. However, development and characterization of this technology is ongoing. New approaches to the FSCV waveform, instrumentation design, reference electrode, surface treatments, and signal processing are all potential areas of future exploration for optimization of BDDMEs.

## 5. Concluding Remarks

Loss or dysregulation of NTs underlies the debilitating consequences of neurodegenerative diseases, substance use disorders, and neuropsychiatric illnesses. Improved technologies which can selectively and sensitively interrogate changes in neurochemicals in the brain may open up new understanding and treatment options for these complex disorders. For example, Parkinson’s disease is characterized by motor dysfunction that is associated with loss of DA transmission caused by progressive degeneration of dopaminergic innervation of the caudate and putamen. It has been estimated that at the time of motor symptom emergence, 50–80% nigral DA neurons have been lost and even more pronounced reductions in dopaminergic innervation in the putamen have already occurred [233,234]. Detection of subtle deficits in NT transmission is desirable for the preclinical testing and development of new neuroprotective therapies. Likewise, simultaneous, real-time detection of NTs across many sites could reveal new understanding of the neurochemical dynamics in affective disorders involving multiple brain regions (e.g., depression). A recent study which used FSCV to interrogate serotonin release in three disparate brain regions found evidence of location-specific reuptake profiles [235]. Such knowledge may facilitate the development of better-targeted and more effective drug treatments. Methods for sensing neurochemicals in vivo would ideally be simultaneously sensitive, minimally-invasive, and relatively inexpensive, while allowing detection with high spatiotemporal resolution across multiple brain regions.

CFMEs, in combination with FSCV, have created new opportunities to detect neurochemicals in the intact brain with high spatiotemporal resolution. Nonetheless, limitations remain with the mechanical integrity and chronic sensing performance of CFMEs. Additionally, construction of CFMEs traditionally involves a multi-step, manual process of threading a carbon fiber into a glass capillary, which is then heated, pulled, broken, trimmed, and sealed with epoxy. Each step is prone to breakage and not well-controlled, resulting in variability between electrodes. Diamond-based microelectrodes, as an alternative, offer a wide potential window, low background current, improved mechanical stability, and more flexible fabrication schemes. While biofouling and loss of sensitivity can be significant challenges in vivo, several opportunities exist to further develop and optimize the performance of BDD sensors. Our team is pursuing the development of a new, all-diamond fiber ultramicroelectrode as a next-generation neurochemical sensor. These devices can be batch-fabricated and offer customizable architectures and site layouts, potentially enabling “chemical mapping” of the neural circuitry underlying normal and pathological brain function. Combined with strategies to optimize performance, diamond electrodes are a promising candidate material for neurochemical sensing.

## Figures and Tables

**Table 3:** Comparison of various modification strategies for BDD electrodes.

Electrode	Linear Range/µM	LOD/µM	Sensitivity/µA µM^−1^	NTs/Interferents	Detection Method	Ref.
Anodically treated BDD	1–70	0.05	---	DA/AA	Chronoamperometry	[187]
Anodically treated BDD	1–100	0.27	---	EP/LID	SWV	[188]
Anodically treated BDD	0.05–20	0.05	---	DA/AA	Chronoamperometry	[105]
Plasma treated porous BDD	0.1–100	0.06	1.05–1.14	DA/B6	Differential pulse voltammetry (DPV)	[183]
Porous BDD via SiO_2_ nanofiber template	0.25–10.0	0.2	0.157	DA/AA, UA	CV & DPV	[182]
BDD-coated black Si	0.3/0.5	0.27	9	DA/UA	CV	[186]
Carbon black/Nafion modified porous BDD	0.1–100	0.054	---	DA/AA, UA	CV & SWV	[189]
AuNPs/PANI/BDD	0.15–500	0.03	0.131	DA/AA	CV & SWV	[192]
Au/PE/PS-modified BDD	5–100	0.8	0.059	DA/AA	CV	[190]
SAM/Au/BDD	0.01–10	0.001	0.00026	DA/AA,	CV	[191]
PPA/PTy-modified BDD	≤80	0.05	0.02	DA/AA, UA, L-DOPA	Chronoamperometry	[194]
PDMA film-coated BDD	0.2–2.6	0.06	0.363	DA/AA	CV & SWV	[193]
ECP-UV treated BDD, ultrananocrystalline	---	0.027	---	DA	FSCV (in vivo)	[153]
Alumina-polished BDD	0.5–1	0.5	---	5-HT	CV	[195]

## Data Availability

The data presented in this study are available on request from the corresponding author. The data are not yet publicly available as preliminary examples to establish proof-of-concept.
